# Magnetic separation of general solid particles realised by a permanent magnet

**DOI:** 10.1038/srep38431

**Published:** 2016-12-08

**Authors:** K. Hisayoshi, C. Uyeda, K. Terada

**Affiliations:** 1 Institute of Earth and Space Science, Graduate School of Science, Osaka University, Japan

## Abstract

Most existing solids are categorised as diamagnetic or weak paramagnetic materials. The possibility of magnetic motion has not been intensively considered for these materials. Here, we demonstrate for the first time that ensembles of heterogeneous particles (diamagnetic bismuth, diamond and graphite particles, as well as two paramagnetic olivines) can be dynamically separated into five fractions by the low field produced by neodymium (NdFeB) magnets during short-duration microgravity (*μg*). This result is in contrast to the generally accepted notion that ordinary solid materials are magnetically inert. The materials of the separated particles are identified by their magnetic susceptibility (χ), which is determined from the translating velocity. The potential of this approach as an analytical technique is comparable to that of chromatography separation because the extraction of new solid phases from a heterogeneous grain ensemble will lead to important discoveries about inorganic materials. The method is applicable for the separation of the precious samples such as lunar soils and/or the Hayabusa particles recovered from the asteroids, because even micron-order grains can be thoroughly separated without sample-loss.

Translations were recently observed for single diamagnetic particles released in an area of a monotonically decreasing field for the purpose of detecting the values diamagnetic susceptibility (χ_DIA_) per unit mass in a small sample; the particles translated freely in a diffuse area in *μg* conditions^1^. It was proposed that χ_DIA_ could be obtained regardless of how small the mass (*m*) of the sample is because the terminal velocity (*v*_T_) of the particles translating in an area outside the field was uniquely determined by χ_DIA_ and by the field intensity at the initial sample position. This relationship was deduced using an energy conservation rule. In a commercial device, the interference of a sample holder and the difficulty in measuring *m* prevent the measurement of χ_DIA_ in a small sample.

In the present report, translation was simultaneously performed on multiple particles for the first time, and we examined whether grains composed of weak magnetic (i.e., diamagnetic or paramagnetic) material could be separated in a simple manner without using a high-field generator. Solid samples are frequently obtained as aggregates of heterogeneous grains^2^,^3^. Although advanced microprobe technologies have enabled aggregate sections to be surveyed *in situ* with high spatial resolution, it is difficult to conclude from these analyses whether the minor particles included in the sample were identified without omission; that is, new categories of minor material phases in the above-mentioned grain aggregates may remain undiscovered. In such cases, it is desirable to separate the grain ensemble into groups of different materials before performing refined analyses. This method of pretreatment is already established in the field of organic analysis using chromatography^4^. In the same manner, a simple method to separate all types of solid particle with fairly good precision is highly desired.

Magnetic separation has been conventionally used to collect materials that are either ferromagnetic, ferrimagnetic or strongly paramagnetic. The dynamic motion of weak magnetic materials generally require strong field intensities above 10 Tesla to be produced using a specific field generator. High-field laboratories have observed that field-gradient forces can cause magnetic levitation of diamagnetic materials^5^,^6^,^7^. Levitation was also realised on a human fingertip by using a small NdFeB magnetic block^8^. Methods to detect the χ_DIA_ of a small particles using the high-field conditions were proposed^9^,^10^. These measurements were performed in the presence of a viscous drag that was caused by the surrounding medium in terrestrial gravity conditions. By applying a static field produced by a strong magnetic field, preferential alignment of magnetically stable axes was realised on an ensemble of diamagnetic molecules^11^ and microparticles^12^,^13^. However, it was later reported that in most micron-sized crystals, the minimum field intensity required to achieve alignment was obtainable using an ordinary electromagnet (i.e., B < 2 Tesla)^14^,^15^.

The 3 diamagnetic samples were cut from high quality synthetic blocks (purity: 99.99 wt%). Using the magnetic and chemical analyses performed in previous studies^1^, we confirmed that the χ_DIA_ values of these materials were consistent with published values^16^. The two olivine samples were products of San Carlos (New Mexico) and Mogok (Myanmar). The paramagnetic susceptibility (χ_PARA_) values of these two samples were 1.26 × 10^−5^ emu/g and 1.39 × 10^−6^ emu/g, as measured using a vibration magnetometer (VSM). To separate the sub-millimetre-sized particles, the apparatus that was previously developed to detect the χ_DIA_ values of a small sample^1^ was modified, as shown in the lower portion of Fig. 1. In this experiment, a monotonically decreasing field distribution was produced along the +x-axis by a small NdFeB circuit (B ≦ 0.6 T). Two collecting plates (A and B) were placed in the translating areas to examine magnetic separation. This system was enclosed in a glass tube, and the inner pressure was reduced to ~100 Pa to eliminate the effect of air resistance. The translating samples were observed by a hi-speed camera (CASIO EX-F1, Japan) placed outside of the glass tube. The above setup was installed in a drop box (40 cm × 30 cm × 22 cm), which was used in a short shaft (*μg* duration <0.5 s)^1^.

The relationship between the velocity and the observed position of the translated particles is shown in Fig. 1. Here, note that the olivine samples, which are paramagnetic, have negative velocity (i.e., the sample translated in the –x direction). The ensemble of particles was initially set at the position x = 0, where the field intensity (*B*(0)) was 0.30 T. As observed from the isochrones shown in the Fig. 1, the extent of particle translation positively correlated with the |χ_DIA_| and |χ_PARA_| values of the particle. The terminal velocity (*v*_T_) of the diamagnetic particles measured in an area outside the field increased with the |χ_DIA_| value. As shown in Fig. 2, the translated particles were all recovered on the two collecting plates as different groups of materials. The field-gradient force is commonly used to attract particles composed of ferromagnetic or ferrimagnetic (and strongly paramagnetic) materials. Field-induced separations have also been obtained for magnetized DNA^17^. In addition, magnetic forces have been used to develop an *in-vivo* drug-delivery system^18^ by attaching a magnetic microbead to the drug particle. As shown in Fig. 1, we were able to demonstrate that simultaneous translation and separation of various weak magnetic particles can be achieved without using magnetic beads.

As observed in previous studies, the translations of weak magnetic grains released in a monotonically decreasing field were well expressed by an energy conservation rule^1^ described as,


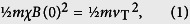


where the initial grain velocity is assumed to be negligibly small. This equation shows that the magnetic potential of a particle at initial sample position is completely converted into the kinetic energy when the particle is outside of the magnetic field. From the above equation, χ_DIA_ of the diamagnetic particles is directly calculated as


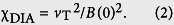


The χ_DIA_ values of individual particles are obtained by inserting the *v*_T_ values determined in Fig. 1 and the *B*(0) value measured before the experiment into equation (2)^1^.

In the case of paramagnetic particles, χ_PARA_ values were estimated using a previously reported energy conservation rule (equation 1) in ref. 1 because the translation of the particle towards the NdFeB plate (S pole) was due to the attractive magnetic force and *v*_T_ was immeasurable. Hence, the value of χ_PARA_ was obtained from the numerical *v*(*x*)-*B*(*x*) relationship that was determined from the numerical data shown in Fig. 1. As shown in Fig. 3, the calculated χ_DIA_ and χ_PARA_ values were consistent with their expected values for the five materials studied in this present report. The numerical range of the measured χ_DIA_ values overlaps with that of published values for existing solid materials^16^. As for paramagnetic materials, the Fe concentrations are below 10 mol% in most of the materials produced in nature; San Carlos olivine is renowned as a major component of the Earth’s upper mantle. The reproducibility of the separation experiment was confirmed by repeating the observation several times in the same conditions (Trail 1 ~ 4 in Fig. 3).

The mass-independent property of magnetic translation described in equation (1) is an essential factor for achieving separation; that is, when a solid particle is released in an area of common *B*(*x*) distribution with a small initial velocity, its position and velocity during translation are uniquely determined by its magnetic susceptibility^1^. Accordingly, a grain ensemble released at a single position in a monotonically decreasing field distribution is separated into groups of different materials as translation proceeds. Furthermore, by comparing the obtained values of χ_DIA_ with published values^16^ for individual particles, the material of the particles can be easily identified without consuming or wasting the sample^1^,^19^. Such identification is possible for any solid material because Langevin-type diamagnetic magnetization is mainly determined by the spatial distribution of the localised electron density in a solid^16^ and the intrinsic χ_DIA_ value of a material (as listed in Table 1) is uniquely determined by the crystal (or molecular) structure of the particle. Indeed, the consistency between the measured and expected values of χ_DIA_ shown in Fig. 3 indicate that the proposed method of separation (and identification) is generally applicable to solids.

Several methods to resolve grain-aggregate material into single particles have been put into practical use; these include a freeze-thaw method^20^ and a high-voltage discharge method^21^. Hence, by performing the field-induced translation described in Fig. 1 on the resolved grain ensemble, minor materials included in the aggregate sample can be separated with relatively small error. Various important achievements have been made in the field of analytical science by extracting minor organic materials from heterogeneous organic solutions using chromatography pretreatment^4^ because this method can separate and identify most existing organic molecules. The method developed in the present work has a comparable significance to the chromatography separation because extraction of new solid phases from heterogeneous grain ensembles using this new method could lead to many important discoveries about inorganic grain materials.

In the investigation of heterogeneous samples, the existence of important minor phases was frequently predicted by theoretical consideration, thereby promoting their subsequent discovery^2^,^3^; however, a hypothesis free survey of the aggregate sample is also necessary for a complete understanding of the process by which a heterogeneous material is formed. The conventional methods of grain separation, such as the heavy media separator, are not frequently used for analytical pretreatment because of their complexity. In addition, the accuracy of conventional methods is not sufficient for refined analysis of individual particles.

The proposed setup is applicable for use in industrial settings (for example, in resource explorations and in the recycling of industrial waste) because it can be directly applied to accumulate and purify various types of resource that are provided in the form of small particles. Note that the efficiency of the above-mentioned methods to resolve grain aggregates into single materials is not guaranteed for some of the heterogeneous materials in general, and it is necessary to develop the technique of resolving the starting material, on a case-by-case basis, when applying this method of magnetic separation to heterogeneous materials. This application is promising because, by using the setup of Fig. 1, magnetic separation of the grain ensemble is easily performed at a low cost in an ordinary facility that dos not have a high-field generator. In other words, the field-induced translation of the sub-millimetre sized particles is realised by a permanent magnet using a short drop shaft^1^,^19^. Note that the small NdFeB circuit was capable of producing a field gradient of 625 G/cm, which was large enough to complete the required translation (several centimetres) within a short time (<1 s). Provided that the spatial resolution for observing translating particles is improved by a refined microscopy system, the separation of micron-sized (or submicron-sized) grains can be realised by modifying the present setup^1^.

Also note that the separation and identification of weak magnetic particles could be used in outer space. For example, in a mission focused on the icy satellites in the outer solar system^22^,^23^, this method could be applied to identify particles, such as volatile solids, silicate and metal, collected at the mission site. According to the data book^16^, the χ_DIA_ values of volatile solids are below −4 × 10^−7^ emu (i.e., H_2_O: −7 × 10^−7^ emu/g, CO_2_: −5 × 10^−7^ emu/g and CH_4_: −8 × 10^−7^ emu/g), whereas most values reported for organic and silicate materials are higher than −4 × 10^−7^ emu (see Table 1)^16^. Therefore, by measuring the quantity of the separated particles using their χ_DIA_ values, the abundance ratio between the volatile, metal and silicate materials can be estimated at the site of mission without the need for samples to return to Earth. As shown in Fig. 3, the present accuracy of the values of the susceptibility obtained from the translation is about 10%. In order to separate SiO_2_, Al_2_O_3_ or CaCO_3_ grain mixture, further improvement of the accuracy will be required in advanced studies (e.g., by reducing the gradient of the monotone decreasing field, which reducing field inhomogeneity).

In future work, field-induced translation could be used to precisely control the position of a substance that is released into the limited experimental area in an orbital laboratory; a reliable technique of position control is required in space technology. Electrostatic suspension is presently considered as a promising method for such purposes, although it requires a diffuse condition to avoid its electric discharge^24^, and relatively large equipment to provide a high-voltage electric supply. Based on the present report, it is expected that the position of an ordinary solid could easily be controlled at normal pressure by changing the spatial distribution of the static magnetic field. Finally, in the diffuse conditions of outer space, the effectiveness of equation (1) would be more efficient because the effects of viscous drag, friction and gravity are negligible. Note that both magnetic fields and dust particles are omnipresent in regions of galactic space that have been studied^25^,^26^,^27^. Even if the field intensity is low, the long *μg* duration in space might allow specific translations of solid particles, enabling chemical fractionation of interstellar molecular cloud. The mass independent characteristics of field-induced translation have also been experimentally confirmed for ferromagnetic and ferrimagnetic grains^28^; i.e. iron, nickel and ferrite. Hence, by optimising the design of the field distribution apparatus, the proposed method of material separation can be realised for all categories of magnetic materials.

## Methods

**Figure 1:**
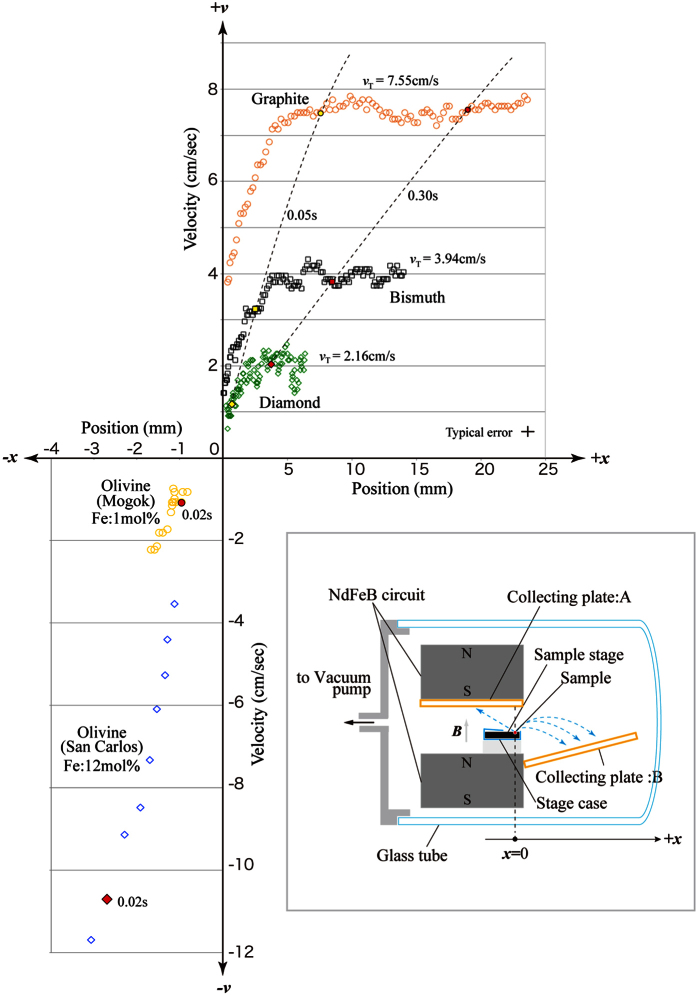
The relationship between velocity (*v*) and sample position (*x*) of sub-millimetre particles observed during translation induced by a magnetic field. A schematic view of the experimental setup is shown in the inset. An ensemble of particles belonging to 5 different materials was released in a diffuse area of a static field that monotonically decreased along the *x*-axis. The *v*-*x* relations were obtained from time-sequential photographs taken from a direction perpendicular to the *x*-axis. The broken curves in the *v*-*x* relationships describe the isochrones of sample position at *t* = 0.05 s and 0.30 s; here, the *μg* condition began at *t* = 0. Note that the 2 olivine samples, which are paramagnetic, have negative position and velocity. At the beginning of the *μg* condition, the sample stage set inside the stage holder was levitated by approximately 0.5 mm, which was effective in releasing the grains with negligible initial momentum in the ±*x* directions; in general, it is technically difficult to release a substance in a diffuse area in *μg* conditions^1^,^19^.

A schematic view of the experimental setup is shown in Fig. 1. Immediately after beginning the *μg* condition, the diamagnetic sample stage set inside the stage-holder was levitated by approximately 0.5 mm, which was effective in releasing the sample grains in a diffuse area of *μg* with negligible initial momentum in the ±x directions; the stage was spontaneously levitated by a small field gradient applied in the +z direction. In previous studies, it was technically difficult to release a substance in a diffuse area in *μg* conditions^1^,^19^. An ensemble of sample grains with different magnetic properties, as described in the text, were released in a position where the static field monotonically decreased along a +x-axis. The *v*-*x* relations shown in Fig. 1 were obtained from time-sequential photographs taken by the hi-speed camera in the +y-axis direction.

**Figure 2:**
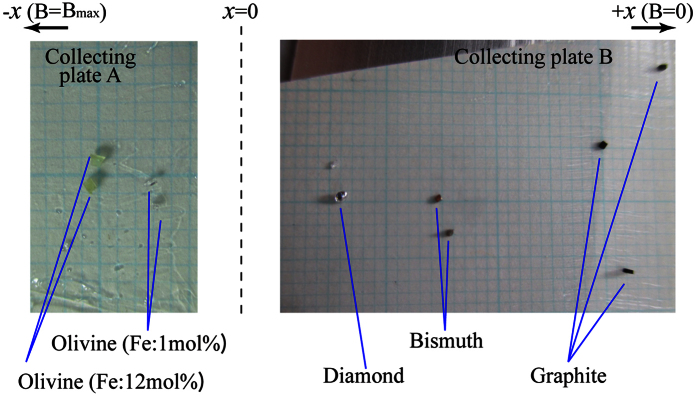
Photographs of the collecting plates A and B that recovered the paramagnetic and diamagnetic particles after magnetic separation. The original positions of the two collecting plates in the experimental setup are described in the inset of Fig. 1. To quantitatively distinguish the positions of the particles, sectional papers were attached to the plates, of which the smallest scale was 1 mm. Before the experiment, the sectional papers were coated with silicone grease to fix the positions of the recovered particles to the paper. In order to reduce the affect of Coulomb force interaction between the grains as much as possible, we neutralized the grain ensemble before the experiment.

The experimental setup described in Fig. 1 was installed in a wooden drop box that was attached to the ceiling of a short drop shaft using an electromagnet. This shaft had a length of 1.5 m^19^. The free-fall of the drop box started shortly after the power supply of the electromagnet was shut down. The duration of the *μg* condition was approximately 0.5 s. To examine the separation of the grains as shown in Fig. 2, the two collecting plates were removed from the experimental setup after the *μg* experiment.

## Additional Information

**How to cite this article**: Hisayoshi, K. *et al*. Magnetic separation of general solid particles realised by a permanent magnet. *Sci. Rep.* **6**, 38431; doi: 10.1038/srep38431 (2016).

**Publisher's note:** Springer Nature remains neutral with regard to jurisdictional claims in published maps and institutional affiliations.

## Figures and Tables

**Figure 3:**
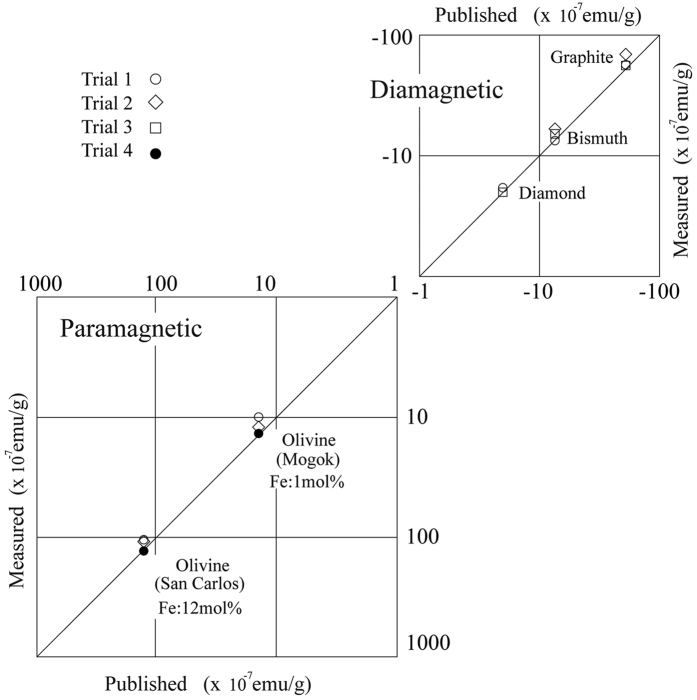
Experimental and published χ_DIA_ values are compared for three diamagnetic materials in the upper portion. The published χ_DIA_ values of existing materials range from 1 × 10^−7^ to 55 × 10^−7^ emu/g^16^, as listed in Table 1; this range nearly overlaps with the range of data in this figure. In the lower portion, the experimental χ_PARA_ values are compared with the values measured by the VSM method (see the main text).

**Table 1:** Published χ_DIA_ values of solid materials^16^.

Material^16^	χ_DIA_ x10^−7^(emu/g)
Graphite: C	−52.0
Bismuth: Bi	−13.4
Methane: CH_4_	−8.00
Anthracene: C_14_H_10_	−7.35
Naphthalen: C_10_H_8_	−7.08
Water: H_2_O	−7.02
Diamond: C	−5.88
Carbon dioxide: CO_2_	−4.77
Cellulose: (C_6_H_10_O_5_)_n_	−4.2
Enstatite: MgSiO_3_	−4
Qurrtz: SiO_2_	−3.7 ~ −4.7
Alumina: Al_2_O_3_	−3.63
Calcite: CaCO_3_	−3.55
Forsterite: Mg_2_SiO_4_	−3.3
Silicon carbide: SiC	−3.19
Gold: Au	−1.42
